# Determining the Incidence of Acute Kidney Injury Using the RIFLE Criteria in the Medical Intensive Care Unit in a Tertiary Care Hospital Setting in Pakistan

**DOI:** 10.7759/cureus.4071

**Published:** 2019-02-13

**Authors:** Syed Waqar Hussain, Aayesha Qadeer, Kamran Munawar, Muhammad Shoaib Safdar Qureshi, Muhammad Tariq Khan, Azmat Abdullah, Sheher Bano, Zahid Siddique Shad

**Affiliations:** 1 Internal Medicine, Khan Research Laboratories Hospital, Islamabad, PAK; 2 Internal Medicine, Shifa International Hospital, Islamabad, PAK

**Keywords:** : akin, kdigo, rifle, acute kidney injury, oliguria, end stage renal disease, aki

## Abstract

Background

Acute kidney injury (AKI) is a frequently encountered clinical condition in hospitalized patients, particularly those admitted to intensive care units (ICU). AKI has its systemic sequelae and contributes to the morbidity of underlying diseases.

Methods

This descriptive case series aimed to determine the frequency of acute kidney injury in critically ill patients admitted to the ICU at Shifa International Hospital, Islamabad, according to the RIFLE (risk, injury, failure, loss, and end-stage) criteria. A total of 124 patients were enrolled in this study. RIFLE criteria were applied to determine the frequency of AKI in critically ill patients.

Results

The frequency of AKI was 68.55% and mortality was 18.55%. The severity of AKI was found to be significantly associated with mortality (p < 0.001).

Conclusion

AKI is very common in critically ill patients and contributes to the mortality and morbidity of the patients. Early identification of AKI can reduce mortality in critically ill patients.

## Introduction

The term acute kidney injury (AKI) encompasses a wide spectrum of disease states, ranging from mild injury characterized by temporary derangement of renal functions at one end to complete loss of renal function at the other end as a result of a severe insult to the renal function [1]. Known to account for 5%-7% of all hospitalized patients, the incidence of acute kidney injury in intensive care units (ICU) patients is almost 20%-50% [2]. AKI is both a cause of and a life-threatening complication of critical illnesses with increased immediate and late morbidity and mortality [3-5].

The criteria used to define acute kidney injury have evolved over a period of time, from relying upon simple markers of renal function, such as serum urea and serum creatinine levels and decreased urine output, to complex definitions, such as the RIFLE (risk, injury, failure, loss, and end-stage) definition, the AKIN (acute kidney injury network) definition, and the KDIGO (kidney disease improving global outcomes) definition [6-10].

The RIFLE classification of acute kidney injury relies on urine output and serum creatinine levels to predict the severity of acute kidney injury in an individual in terms of risk, injury, or failure to describe two possible outcomes, i.e., loss of renal function and end-stage renal disease. However, one has to know the baseline serum creatinine to apply this classification. The RIFLE classification has been found to be a valid classification system for predicting acute kidney injury in hospitalized patients as well as for the prediction of prognosis in such patients [11-15].

With this knowledge, we decided to determine the incidence of acute kidney injury in patients admitted to the intensive care unit at Shifa International Hospital, Islamabad, using the RIFLE criteria and to determine the outcome in terms of length of stay and mortality.

## Materials and methods

After approval from the institutional review board (IRB) at Shifa International Hospital, a prospective study designed as a case series was conducted from July 2017 to January 2018. This was at the medical intensive care unit (ICU), Shifa International Hospital, Islamabad. For the purpose of this study, acute kidney injury was defined as an abrupt (occurring within 48 hours of admission to the medical ICU) reduction in kidney function. The reduction in kidney function was defined in terms of “either an absolute increase in serum creatinine of more than or equal to 0.3 mg/dl, a percentage increase in serum creatinine of ≥ 50% (1.5-fold from baseline), or a reduction in urine output (documented oliguria of <0.5 mL/kg/hr for >6 hrs)” [16]. Serum creatinine was measured using spectrophotometry at the pathology laboratory of Shifa International Hospital and the glomerular filtration rate (GFR) for each patient was determined using the Cockcroft-Gault formula. The outcome of the study was measured in terms of length of stay (days) in the medical ICU and mortality. For the purpose of this study, a sample size of 124 was arrived at using the World Health Organization (WHO) sample size calculator software with the following assumptions: Confidence Interval (CI) 95%, anticipated proportion of acute kidney injury in critically ill patients (p): 8.8%, and absolute precision (d): 5% [14].

The consecutive non-probability sampling technique was used to complete the sample size. All patients admitted to the medical ICU of Shifa International Hospital, Islamabad, except those with chronic kidney disease, were enrolled in this study. Prior approval was obtained from the hospital ethics committee and informed consent was obtained from either the patients or their family members, where applicable. Serum levels of creatinine, as well as urine output, were monitored during the first 24 hours stay at the ICU. Urine output was measured specifically at 6 hrs, 12 hrs, and 24 hrs, in addition to the hourly monitoring of urine output. Then, using the creatinine increase/calculated GFR and urine output, the RIFLE category was assessed for each patient. Based on the RIFLE criteria, the outcome in terms of ICU mortality and ICU length of stay was recorded by the trainee researcher and findings were recorded in the proforma. The collected data were entered into and analyzed using SPSS 16 (IBM Corp., Armonk, NY, US). Descriptive statistics were computed, as mean and standard deviations were presented for age and duration of ICU stay. Frequencies and percentages for variables like gender, AKI using the RIFLE criteria, the RIFLE class, and mortality were calculated. Analysis of variance (ANOVA) was used for the comparison of quantitative variables (length of stay) and Chi-square was used to determine differences in the outcome of RIFLE categories. The RIFLE criteria are explained in Table 1.

**Table 1:** RIFLE criteria RIFLE: risk, injury, failure, loss, and end-stage; GFR: glomerular filtration rate

	GFR Criteria	Urine Output Criteria
RISK	Increased Cr _Serum_ x 1.5 or GFR decreased > 25%	Urine output < 0.5 ml/kg/h x 6 hours
INJURY	Increased Cr _Serum_ x 2.0 or GFR decreased > 50%	Urine output < 0.5 ml/kg/h x 12 hours
FAILURE	Increased Cr _Serum_ x 3.0 or GFR decreased > 75% Or Cr _Serum_ >4 mg/dl	Urine output < 0.3 ml/kg/h x 24 hours or anuria x 12 hours
LOSS	Persistent Acute Kidney Failure = Complete loss of kidney functions> 4 weeks	
ESRD	End Stage Renal Disease (ESRD) Complete loss of kidney function> 3 months	

## Results

There were 77 (62.1%) males and 47 (37.9%) females with the mean ± SD age of study participants as 55.71 ± 18.06 years (age range from 18 to 97 years). The mean length of ICU stay was 4.73 ± 3.75 days (with a range of 1-20 days). Fifty-eight (46.8%) of the patients stayed in the ICU for one to three days (Table 2).

**Table 2:** Length of ICU stay ICU: intensive care unit

Length of ICU stay	Percentage
1-3 Days	46.8
4-6 Days	30.6
7-9 Days	15.3
10-12 Days	1.6
13-15 Days	2.4
16-18 Days	1.6
19-21 Days	1.6

The commonest reasons for admission to the ICU were pulmonary disease (n= 29; 23.4%), cardiac disease (n=27; 21.8%), and sepsis (n=25; 20.2%). Other reasons for admission to the ICU were central nervous system (CNS) disease (n=21; 16.9%), poisoning/overdose (n=6; 4.8%), hepatic disease (n=5; 4%), malignancy (n=4; 3.2%), and miscellaneous causes (n=7; 5.6%) (Table 3).

**Table 3:** Primary diagnosis of ICU patients ICU: intensive care unit

Diagnosis	Frequency	Percentage (%)
Cardiac	27	21.8
Sepsis	25	20.2
Pulmonary	29	23.4
Malignancy	4	3.2
Poisoning/overdose	6	4.8
CNS	21	16.9
Hepatic	5	4.0
Misc	7	5.6
Total	124	100.0

The frequency of AKI in our study was 68.54%, with 85 patients developing AKI during their stay in the ICU. Of these 85 patients, 34 (40%) were categorized as R (risk), 38 (44.70%) as I (injury), and 13 (15.3%) as F (failure) (Table 4). Twenty-three (18.5%) patients died during the period of this study.

**Table 4:** RIFLE severity category RIFLE: risk, injury, failure, loss, and end-stage

RIFLE Class	Frequency	Percentage (%)
Risk	34	40
Injury	38	44.7
Failure	13	15.3
Total (n)	85	100.0

In terms of patient outcome with reference to mortality, of the patients falling in class R, 85.29% (n=29) were transferred out of the ICU while 14.7% (n=5) of them expired while in class I, 84.2% (n=32) got transferred out, and 15.78% (n=6) expired. In class F, 38.46% (n=5) patients were transferred out whereas 61.53% (n=8) patients expired (Table 5). This shows that as the severity of renal failure increases, the patient mortality also increases since the highest mortality of AKI patients is seen in those falling in class F.

**Table 5:** RIFLE category outcome cross-tabulation AKI: acute kidney injury; ICU: intensive care unit; RIFLE: risk, injury, failure, loss, and end-stage

RIFLE Class	Outcome		Total
	Transferred out of ICU	Expired	
Risk	29 (85.29%)	5 (14.7%)	34
Injury	32 (84.2%)	6 (15.78%)	38
Failure	5 (38.46%)	8 (61.53%)	13
No AKI	35 (89.7%)	4 (10.3%)	39
Total	101	23	124

Only four (10.3%) patients with no AKI expired as compared to 5 (14.7%) in R, six (15.8%) in I, and eight (61.5%) in F. When the outcome was cross-tabulated with the RIFLE category, a statistically significant association was found between the severity of AKI and mortality (p < 0.001). There was, however, no difference in length of ICU stay and RIFLE class.

## Discussion

Acute kidney injury is commonly diagnosed in medical ICUs across the world and is characterized by morbidity related to its systemic sequelae and its extra-renal organ system effects [17-19]. Additionally, the presence of AKI adversely affects the prognosis of a disease in both the short and long terms [20]. Whereas the incidence of AKI in the general population is considered to be low, its incidence in hospitalized patients is much greater, and in ICU patients, it can be as much as 50% [21-24]. With the introduction of classification systems, such as AKIN and RIFLE, the incidence of AKI has increased, partly because of increased identification, so much so that up to 70% of ICU patients have been reported to have some sort of AKI or renal dysfunction [25]. AKI has a mortality rate of 20%-90% [26].

The incidence of AKI in our study was 68.55% with a mortality rate of 18.55%. A variable incidence of AKI has been reported in the literature. For example, a study from the Netherlands reported a 35% incidence of AKI according to the RIFLE criteria in critically ill patients [6]. The authors noted that the reported incidence of AKI in critically ill patients depends on a number of factors such as the definition of AKI used, the used of baseline or reference serum creatinine, and the inclusion/omission of urine output in the staging of AKI.

Another study reported that the frequency of AKI was 57.3% in critically ill patients. The authors reported that the increasing severity of AKI was associated with increased mortality in the hospital. Our results point toward similar findings [17]. A study from the US reported that AKI was diagnosed in 52.6% of ICU patients and AKI was independently associated with a longer duration of hospital stay and short-term (in hospital/ICU) and long-term mortality (after discharge from the hospital) [27].

The authors also identified certain factors, such as hypotension, the severity of underlying illness, diabetes mellitus, and surgical reasons for hospital admission, as predictors for AKI. In our study, the most common cause of ICU admission was pulmonary and the other common causes were cardiac and sepsis, whereas in most international data, sepsis is the most common cause of ICU admission and, in turn, an important contributory factor for the development of AKI in the ICU [28].

Our study had some limitations. First, it included only 124 patients, which is a small sample size for being a powerful study. Second, despite the fact that we had hourly monitoring of the urine output, we didn’t have data regarding diuretic usage in patients, which can influence the urine output. Third, the patients included did not have a longer follow-up so AKI was only categorized as R, I, and F and further classification into loss of kidney function and end-stage kidney disease was not possible. Despite these limitations, our study had some strength. First, it is providing local data in terms of AKI classification and severity. Second, both creatinine and urine output criteria were used to define and categorize AKI.

## Conclusions

AKI is very common in critically ill patients and contributes to the mortality and morbidity of the patients. Early identification of AKI can reduce mortality in critically ill patients.
